# Super Resolution Microscopy Reveals that Caveolin-1 Is Required for Spatial Organization of CRFB1 and Subsequent Antiviral Signaling in Zebrafish

**DOI:** 10.1371/journal.pone.0068759

**Published:** 2013-07-09

**Authors:** Kristin A. Gabor, Chad R. Stevens, Matthew J. Pietraszewski, Travis J. Gould, Juyoung Shim, Jeffrey A. Yoder, Siew Hong Lam, Zhiyuan Gong, Samuel T. Hess, Carol H. Kim

**Affiliations:** 1 Graduate School of Biomedical Sciences, University of Maine, Orono, Maine, United States of America; 2 Department of Molecular and Biomedical Sciences, University of Maine, Orono, Maine, United States of America; 3 Department of Physics and Astronomy, University of Maine, Orono, Maine, United States of America; 4 Department of Biological Sciences, National University of Singapore, Singapore; 5 Department of Molecular and Biomedical Sciences, North Carolina State University, Raleigh, North Carolina, United States of America; Institut Curie, France

## Abstract

Understanding spatial distribution and dynamics of receptors within unperturbed membranes is essential for elucidating their role in antiviral signaling, but conventional studies of detergent-resistant membrane fractions cannot provide this information. Caveolae are integral to numerous signaling pathways and these membrane domains have been previously implicated in viral entry but not antiviral defense. This study shows, for the first time, the importance of spatio-temporal regulation of signaling receptors and the importance of the regulation of clustering for downstream signaling. A novel mechanism for virus evasion of host cell defenses is demonstrated through disruption of clusters of signaling molecules organized within caveolin-rich domains. Viral infection leads to a downregulation in Caveolin-1b (Cav-1b), disrupting clusters of CRFB1, a zebrafish type I interferon receptor (–R) subunit. Super-resolution microscopy has enabled the first single-molecule imaging of CRFB1 association with cav-1b-containing membrane domains. Strikingly, downregulation of Cav-1b, the major protein component of caveolae, caused CRFB1 clusters to disperse. Dispersal of CRFB1 clusters led to a suppressed antiviral immune response both *in vitro* and *in vivo*, through abrogation of downstream signaling. This response strongly suggests that CRFB1 organization within cav-1b-containing membrane domains is critical for IFN-mediated antiviral defense and presents a previously undescribed antiviral evasion strategy to alter IFN signaling and the antiviral immune response.

## Introduction

The structure and organization of cellular membranes play important roles in a wide range of biological processes. Caveolae are specialized membrane nanodomains with a distinct Ω-shaped morphology in the membrane. Caveolae may act as signaling platforms by allowing signaling molecules to cluster together within their ordered domains, facilitating interactions among the components [1]. Critical cellular processes associated with caveolae include signal transduction, cholesterol homeostasis, and adaptive immune signaling [2], [3], [4], [5], [6], [7].

Caveolin-1 (Cav-1) serves as one of the structural components of caveolae and also functions as a scaffolding protein that recruits signaling molecules to caveolae [8]. Clustering of proteins in caveolae provides an environment and a mechanism for controlling probabilities of protein interaction and modulating the efficiency of signal transduction. For example, epidermal growth factor receptor (EGFR) has been shown to interact with caveolin [9] and changes in receptor clustering may provide a mechanism for regulating EGFR signaling [10]. In addition, caveolae are exploited by some viruses to initiate infection [11], [12], whereas other viruses enter cells without the involvement of caveolae [13], [14], [15], [16], [17], [18]. For example, Damm *et al.* [15] observed that when introduced to cells devoid of caveolae, SV40 exploits an alternative, cav-1–independent pathway in the absence of caveolae. Ewers *et al*. [18], used transmission electron microscopy to demonstrate that SV40 induced the formation of membrane invaginations in the absence of caveolar coats. It has also been determined that ebola virus can fully infect cell types lacking caveolae [14] and that SARS coronavirus entry was mediated by a clathrin- and caveolae-independent mechanism [16].

Type 1 interferon (IFN) is crucial for initiation of the innate response to viral infection, and knockout studies in mice have shown that disruption of this response renders the host more susceptible to infection. Other studies have demonstrated that mice lacking a functional IFN receptor (IFN-R) were unable to cope with an array of viral infections, including vaccinia virus, vesicular stomatitis virus (VSV), and Semliki Forest Virus [19]. IFN-R knockout mice were highly susceptible to infection with VSV due to high levels of viral replication [20].

The relationship between cell membrane organization and the antiviral immune response is largely unexplored. One of the primary antiviral responses is the generation of IFN, and only recently has the role of lipid rafts in interferon production come under scrutiny [21]. IFN-R and Caveolin-1 have both been found in detergent-resistant membrane (DRM) fractions [21], but have not been observed with sufficient spatial resolution to determine their nanoscale distribution in intact cell membranes. Such evidence could provide critical insights into the spatial and temporal organization of antiviral receptors and nanodomains.

It has previously been shown that zebrafish infected with snakehead rhabdovirus (SHRV) produce an IFN response [22], [23], leading to the binding of IFN to its cognate receptor, IFN-R. The zebrafish IFN-R complex has been recently identified as cytokine receptor family members CRFB1, CRFB2, and CRFB5, which constitute CRFB1/CRFB5 and CRFB2/CRFB5 heterodimers [24], [25]. The Jak-STAT signal transduction pathway is activated upon IFN binding to the receptor and culminates in the expression of IFN-stimulated response element (ISRE) driven IFN-stimulated genes (ISGs) [26]. The IFN pathway and ensuing antiviral response of zebrafish is similar to that described in mammalian systems [24], [25], [27], [28].

Many properties of membrane domains cannot be understood solely from DRM studies [29], because DRMs isolated from cells may not correspond precisely to preexisting rafts in living cells [30]. The small size of caveolae and the spatial proximity of proteins prohibit direct visualization of the dynamic interaction between the host cell membrane nanodomains and antiviral receptors using conventional light microscopy. Fluorescence photoactivation localization microscopy (FPALM) (Figure S1) [31], [32] is a novel, super resolution technique that extends the resolution of optical microscopy below the diffraction limit, which is on the order of 250 nm, allowing for spatial resolution on the scale of 20–40 nm [31], [32], [33]. Three-dimensional FPALM has achieved a lateral resolution of 30 nm and 75 nm axially [34]. FPALM is well suited for investigation, at the single molecule level, of the highly complex molecular structures and mechanisms underlying biological processes.

Few investigators have focused on the role of caveolae in the immune response. Our results suggest that CRFB1 interacts with caveolae and that caveolae may be critical for maintaining spatial organization and clustering of CRFB1 molecules. The present study demonstrates a novel role for cav-1b-containing membrane domains in the zebrafish response to viral infection. We demonstrate that upon virus infection, cav-1 is downregulated, circumventing the host antiviral IFN response. *In vivo* knockdown studies showed that disruption of the IFN response by cav-1 depletion renders the host more susceptible to infection. Using FPALM, we show that cav-1b-containing membrane domains corral CRFB1 molecules together and that this clustering of CRFB1 is critical for a robust antiviral immune response. In addition, we determined that the membrane protein Cav-1 is responsible for maintaining the CRFB1 clustering and that the functional consequence of Cav-1 depletion is CRFB1 dispersion and abrogation of downstream signaling. By gaining an understanding of the complex dynamics of membrane domains and the mechanisms through which viruses modulate their function, we will better understand how viruses evade host antiviral mechanisms and can implement this knowledge to develop more targeted therapeutics.

## Results

### Cav-1b Colocalizes with CRFB1 and Corrals CRFB1 in Membrane Domains

**Figure 1:**
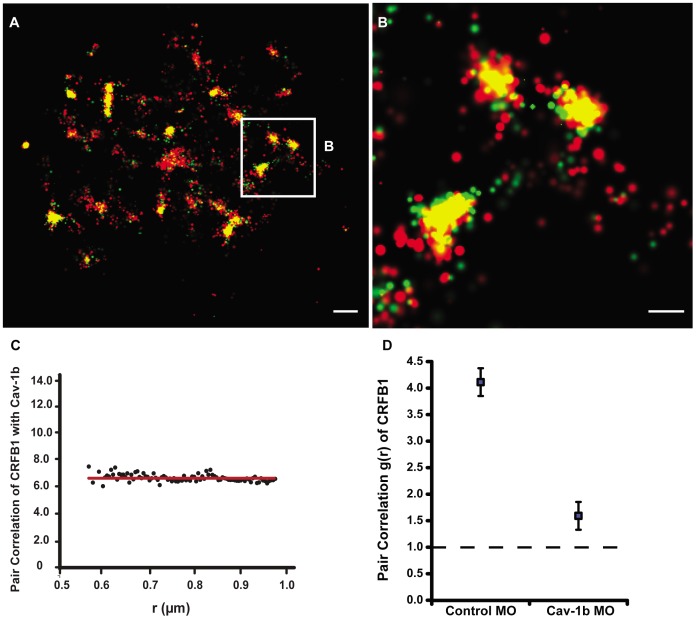
Cav-1b colocalizes with the zebrafish homolog of IFN-R and is positively correlated. ZFL cells (n≥10) were transfected with Cav-1b-PAmCherry (red) and with CRFB1-dendra2 (green). For all images, 60×/1.2 NA magnification. Scale bars, 1 µm. Shown is the plasma membrane of one cell representative of the experiment (**A**) and a magnification (**B**) of the region marked by the white box in A. The image shows that Cav-1b and CRFB1 colocalize in the cell membrane. (**C**) Measurements of Cav-1b and CRFB1 show a positive pair correlation value g(r) greater than one, confirming that the two species are colocalized together. Pair correlation calculations were performed as described in Methods; briefly, g(r) >1 indicates positive correlation/clustering, and g(r) = 1 indicates a random distribution. (**D**) Pair correlation measurements of CRFB1 were calculated for the receptor, control morpholino (MO), and Cav-1b MO. CRFB1 is more prone to random distribution when Cav-1b is knocked down. Error bars SEM (n ≥8 cells).

We investigated the membrane localization of the CRFB1 subunit of the zebrafish IFN-R complex, the components of which are necessary for a functional IFN response in the zebrafish [25]. To test whether CRFB1 localizes to cav-1b-containing membrane domains, FPALM was used to simultaneously image CRFB1-dendra2 and Cav-1b-PAmCherry 24 h after transfection of zebrafish liver (ZFL) cells. ZFL cells express endogenous cav-1b and CRFB1 mRNA (Figure S2a), as do rat liver cells [35], liver sinusoidal cells [36] and primary rat hepatocytes [37]. Figure 1 illustrates that at the surface of a single cell, CRFB1 colocalizes with Cav-1b. Acquisition conditions and more details about FPALM imaging and analysis are described in the Methods section and Figure S1. These data were acquired in the absence of ligand stimulation and show that clusters of Cav-1b molecules are in very close proximity to CRFB1 molecules, and in many instances overlap within the estimated spatial resolution of the technique (∼20 nm). To quantitatively explore this result, pair correlation analysis was performed for CRFB1 and Cav-1b (Figure 1c). Pair correlation between the two species had a g(r) value greater than one, which implies that the two molecules are not randomly distributed, and instead, are colocalized. Additionally, when Cav-1b is knocked down in ZFL cells using a previously characterized morpholino oligonucleotide (MO) [2], the clustering of CRFB1 is significantly decreased, with a more random distribution than observed in controls (Figure 1d).

### Cav-1b is Downregulated by Virus Infection and Cav-1b Morphants show Increased Mortality and Viral Burden

**Figure 2:**
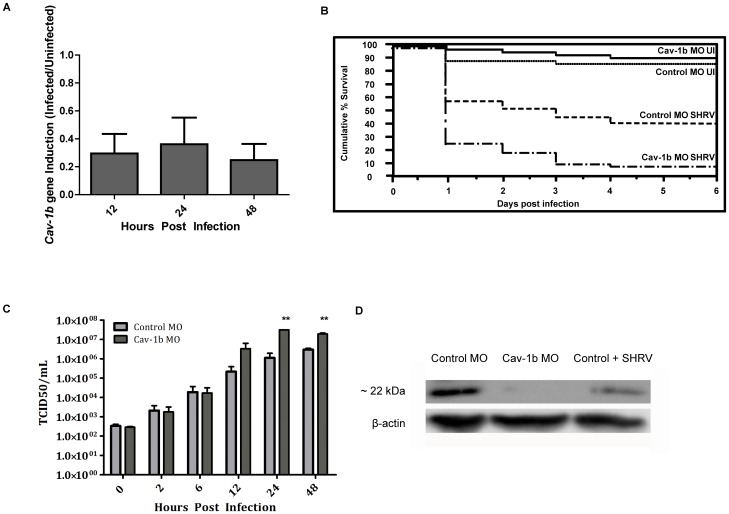
*Cav-1b* expression is modulated during virus infection, and Cav-1b knockdown leaves morphants susceptible to infection. A) Quantitative RT-PCR results revealed fold changes in the expression levels of *Cav-1b* in infected embryos when compared to uninfected embryos. Zebrafish were exposed seven dpf to 1×10^6^ TCID_50_/mL virus. Total RNA was isolated from at 12, 24, and 48 hours post infection and reverse transcribed to cDNA (n = 20 fish per time point). All expression values have been normalized to the zebrafish β-actin gene. Error bars represent SEM of three replicates. B) Zebrafish embryos that were injected with Cav-1b morpholino (MO) to knock down the expression of Cav-1b or control MO were infected 48 hpf with 1×10^6^ TCID_50_/ml virus and monitored for mortality. Results are representative of three separate experiments. Statistical analysis (Wilcoxon test) of the Kaplan-Meier curve was performed (*, p = 0.008). C) Zebrafish embryos that were injected with Cav-1b MO to knock down the expression of Cav-1b or control MO were infected by static immersion 48 hpf with 1×10^6^ TCID_50_/ml virus. The graph indicates that early in infection (0–12 hpi), there is no difference in viral burden between Cav-1b morphants and controls. However, by 24–48 hpi, Cav-1b morphants have a higher viral burden. Figure is representative of three experiments; error bars are standard error of the mean (*, p<0.05). D) Western blot showing efficacy of MO knockdown in zebrafish. Zebrafish embryos from Control and Cav-1b MO, and Control MO with SHRV infection were compared for cav-1b expression at the 72 hpf developmental stage. At this time, infected fish were 24 hpi. Membranes were re-probed with antibody against β-actin to control for protein loading.

The observation of colocalization between cav-1b-containing membrane domains and CRFB1 molecules led to the investigation of whether Cav-1 plays a role in the antiviral response to virus infection, since IFN is a critical component of the innate immune response. The roles of both Cav-1a and Cav-1b in zebrafish development have been previously revealed using MO knockdown technology [2]. Further, the presence of caveolae in zebrafish has been confirmed via electron microscopy [2]. Compared to Cav-1a, Cav-1b in the zebrafish is more similar to Cav-1b in human and mouse, and in previous studies the two isoforms have been shown to have non-redundant roles [2]. Our studies revealed that although *cav-1a* gene expression was also downregulated after SHRV infection (Figure S3a), the effect was not as pronounced, nor was it as long lasting as the downregulation of *cav-1b* gene expression (Figure 2a). Furthermore, when compared to controls, knockdown of Cav-1b resulted in greater mortality than knockdown of Cav-1a after SHRV infection (Figure S3b), leading us to focus our subsequent studies on Cav-1b.

In embryos infected with SHRV, early *cav-1b* gene expression was shown by quantitative RT-PCR to be significantly dampened at 12, 24, and 48 hpi, with a 3.5-, 2.5-, and 3.8-fold decrease in transcript levels compared to controls, respectively (Figure 2a, p<0.05). In order to confirm that during viral infection general suppression of all host gene expression did not occur, zebrafish β-actin primers were used to normalize the initial quantity of RNA, as previously described [38]. To confirm these results, the 18S housekeeping gene was also used to normalize the gene expression in the RT-PCR experiments. The 18S gene has been previously characterized in the zebrafish and shown to be stable during development and across tissue types [22], [23], [39]. The 18S gene was selected due to its high, relatively stable expression levels. If viral infection globally affected gene expression, then 18S would also be influenced. However, similar results (data not shown) were obtained when 18S primers were used to normalize the quantity of RNA.

Knockdown of Cav-1b with MO in embryos has been characterized previously and a reduction in Cav-1b protein levels, as well as a reduction in the number of caveolae domains, was demonstrated [2], [40]. We performed knockdown experiments as described [2], [40] after confirming the amount of MO required to knock down Cav1b. Figure 2d demonstrates that at 24 hpi in control MO-injected embryos Cav-1 protein was still detected, while in Cav-1b MO-injected embryos no Cav-1 protein was found. Western blot analysis of age-matched SHRV infected embryos detected endogenous Cav-1 protein at lower levels than in Control MO embryos, but greater than in the Cav-1b MO samples. This result shows effective knockdown of Cav-1b protein expression by morpholino injection (Figure 2d). These experiments were performed using whole embryo lysates, and so in order to more thoroughly understand the results, we sought to identify tissues in which Cav-1b is expressed. We examined cell type-specific pools of cDNA from adult zebrafish. Of particular interest, cav1b expression was detected in liver, kidney, lymphocyte, and myeloid lineages (Figure S2c). In addition, cav-1b and CRFB1 are also expressed in the liver tissue of embryos when infection studies were performed (Figure S2b).

The Cav-1b MO was used to determine whether the observed disruption of *cav-1b* gene expression would alter the host’s susceptibility to virus infection [2]. Morphant and control embryos were monitored for survival rates and viral burden. In the absence of virus, knockdown of Cav-1b did not affect embryo survival. Kaplan-Meier curves [41] were constructed showing survival of Cav-1b morphants compared to controls after viral challenge, and revealed that Cav-1b morphant embryos exhibited a significant increase in mortality (p = 0.009) compared to the controls (Figure 2b). Uninfected control morphants had low levels of mortality, similar to that of uninjected controls. Cav-1b morphants showed increased mortality throughout the first three days post infection. After just 24 hpi, over 70% of the Cav-1b morphants had succumbed to the infection compared to ∼40% of control infected embryos. Controls and Cav-1b morphants that were uninfected both had ∼90% or greater survival rates. Since the adaptive immune response is not fully developed in zebrafish at the developmental stage selected for these studies [24], [42], [43], the results are due solely to perturbation of the innate immune response.

We sought to determine whether the increase in mortality was a result of increased incidence of viral entry due to Cav-1 knockdown, or reduced ability of morphant embryos to clear the infection. Preliminary studies suggested that SHRV does not utilize cav-1b-containing membrane domains as a means of entry *in vitro* (Figure S4); therefore entry of virus should not be affected by Cav-1 knockdown. Viral burden assays were conducted to determine if disruption of Cav-1b mediated viral entry after infection with SHRV. From 0–12 hpi, no significant increase in viral burden was observed between control and cav-1b MO embryos, which were infected at 48 hpf (and therefore 48 h after MO injection). However, by 24–48 hpi, a significant increase in viral burden (Figure 1c) was measured. Cav-1b morphants showed a 28-fold and 6.5-fold increase in viral titer compared to controls at 24 and 48 hpi, respectively (Figure 2c). The data were significant (two-way ANOVA, p<0.05) and correlated with the increased mortality shown at 24 hpi and 48 hpi in the Cav-1b morphant embryos.

### Disruption of Cav-1b Adversely Affects the IFN Pathway

**Figure 3:**
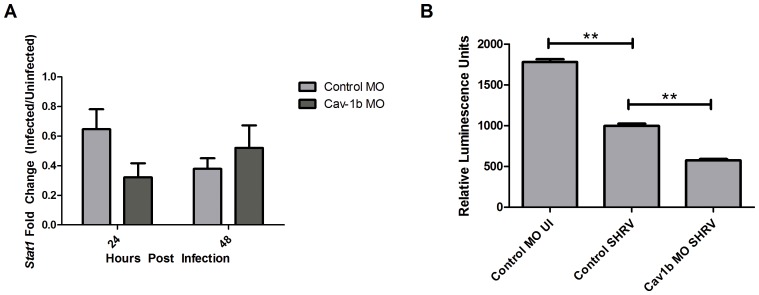
Decrease of Cav-1b expression negatively affects the IFN pathway. A) *Stat1* gene expression was assessed by qRT-PCR in Control MO and Cav-1b MO embryos that were either SHRV infected or uninfected. Total RNA was extracted from 10 fish per treatment, cDNA synthesized and *Stat1* mRNA expression assessed by qRT-PCR 24 hpi. The data are representative of three individual experiments and error bars indicate SEM. Each bar represents the mean fold induction of SHRV-infected embryos over corresponding controls. All expression values were normalized to zebrafish 18s. B) ISRE promoter activity is dampened in Cav-1b knockdown ZFL cells upon SHRV infection. ZFL cells were transfected with 250 ng of zISRE-luc construct along with 250 ng of cav-1b MO or control MO. Twenty four hours post transfection the ZFL cells were infected with SHRV at an MOI of 0.01. Cells were harvested for luciferase measurements 24 hpi. The graph shows relative luminescence units of control uninfected cells compared to cav-1b MO or control infected cells. Error bars are representative of SEM for two experiments. (**, p<0.001).

If cav-1b-containing membrane domains are being used as a platform for immune signaling through the IFN-R pathway, knockdown of Cav-1b should dissipate antiviral signals, such as gene expression of *Stat1* and subsequent induction of ISRE. Transcript levels of *Stat1* were assessed at 24 h in both Control MO and Cav-1b MO embryos with and without SHRV infection. A 1.5-fold (±0.13) decrease was observed in control MO embryos and a 3.1-fold (±0.09) decrease was observed in Cav-1b MO embryos (Figure 3a).

To compare the effect of Cav-1b depletion on the antiviral response to pathogen, we examined control MO cells, Cav-1b MO cells, and control cells after SHRV infection in an ISRE promoter-driven luciferase assay. ZFL cells were transfected with Cav-1b MO or standard control MO, along with an ISRE luciferase construct [44], and subsequently exposed to SHRV (0.01 MOI for 24 h) (Figure 3b). Cav-1b depletion by Cav-1b MO in ZFL cells is shown in Figure S5. SHRV infected cells displayed a significant decrease in ISRE activity compared to control MO samples (two-tailed Student’s t test, p<0.001). Similarly, depletion with Cav-1b MO also resulted in a significant decrease in ISRE activity compared to control MO samples (two-tailed Student’s t-test, p<0.001), mimicking the effect of SHRV infection. A greater reduction in ISRE activity was observed in either SHRV-infected or Cav-1b MO cells when compared to control MO infected cells, a finding that is consistent with the decrease in *Stat1* gene expression shown in Figure 3a.

### Clustering of CRFB1 is Critical for Efficient and Robust Innate Immune Response

We examined whether disrupted IFN signaling resulting from Cav-1b depletion was due to dispersal of CRFB1 molecules corralled by cav-1b-containing membrane domains, or to effects on other antiviral components that could exist within cav-1b-containing membrane domains. Covalent crosslinking studies were performed using bis(sulfosuccinimidyl) suberate (BS^3^) reagent with ZFL cells that were transfected with either Cav-1b MO or standard control MO and subsequently crosslinked. The crosslinking reagent was employed to “rescue” the dispersal of CRFB1 that results from cav-1b disruption. If Cav-1b depleted cells with crosslinked CRFB1 molecules were able to produce an antiviral response, this would indicate that Cav-1b depletion and subsequent dispersal of receptor molecules was directly responsible for the abrogated antiviral response.

**Figure 4:**
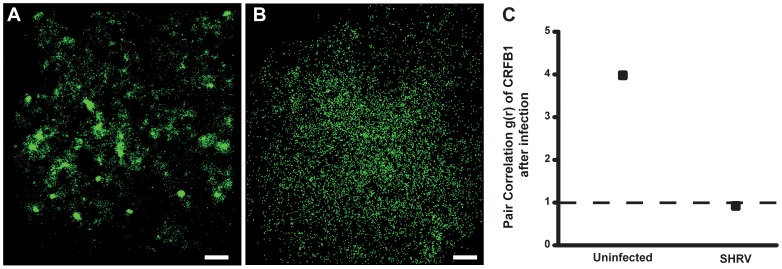
CRFB1 becomes dispersed as a result of whole virus infection *in vitro*. ZFL cells were infected 24 h post transfection and fixed prior to imaging. For all images, 60×/1.2 NA magnification. Scale bars, 1 µm. Shown for each part is the surface of one cell representative of the experiment. A) Uninfected cells overexpressing CRFB1 demonstrate that the receptor exists in clustered patches indicative of caveolae. B) Cells infected with SHRV demonstrate that CRFB1 becomes dispersed as a result of virus infection by 24 hpi. C) Pair correlation analysis confirms that compared to uninfected cells, CRFB1 becomes dispersed after infection. Values of g(r) in cells with SHRV infection are considered to be random in comparison to values in cells that remain uninfected (n≥8 cells per treatment).

**Figure 5:**
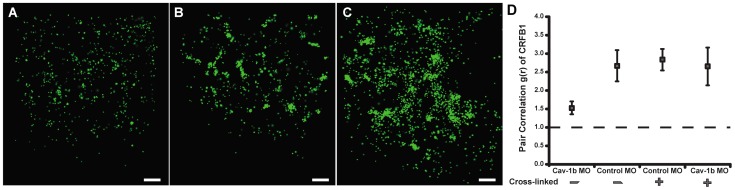
Crosslinking CRFB1 keeps receptor molecules clustered despite caveolin depletion. ZFL cells were co-transfected with MO and expression plasmid via nucleofection and allowed to recover/adhere to cell culture plates for ∼6 hr prior to addition of crosslinking reagent. The crosslinking reaction was performed according to the manufacturer’s procedures. Cells were subsequently replenished with media and returned to the incubator for 24 hr post crosslinking. Scale bars, 1 µm. A) Cells transfected with Cav-1b MO/CRFB1 without crosslinking treatment show dispersed receptor molecules. B) Cells transfected with Control MO/CRFB1 with crosslinking clearly show clustered receptor molecules. C) Cells transfected with Cav-1b MO/CRFB1 with crosslinking. This demonstrates that despite depletion of Cav-1b, receptor molecules remain clustered. D) Pair correlation analysis confirms that with crosslinking, CRFB1 remains clustered despite Cav-1b depletion. Values of g(r) in cells with Cav-1b KD are similar to that for Controls (n≥8 cells per treatment).

FPALM imaging demonstrated that infection of ZFL cells with virus resulted in dispersion of CRFB1 molecules (Figure 4). Similar numbers of CRFB1 molecules are seen in the uninfected cell (12,251) compared to the infected cell (11,358), which indicates that there is no overall loss of surface CRFB1 as a result of infection. These results demonstrate that virus infection leads to dispersal of IFN receptors. Control MO with crosslinking treatment yielded CRFB1 molecules that remained clustered together, while Cav-1b depletion without crosslinking treatment yielded CRFB1 molecules that were dispersed (Figure 5). Pair correlation analysis quantitatively confirmed the result of our FPALM images, showing that with Cav-1 depletion and crosslinking treatment, the receptors remained clustered (Figure 5d).

**Figure 6:**
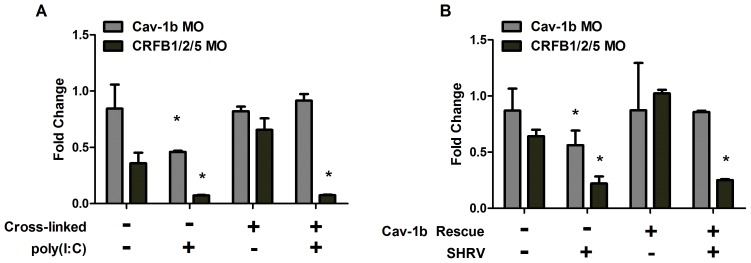
MxA expression is retained with rescue of Cav-1b depletion. ZFL cells were transfected and rescued as described in Methods. Shown is the fold difference in gene expression of MxA, an interferon stimulated gene. MxA transcript levels in Cav-1b depleted cells treated with crosslinking reagent (A) or rescued with cav-1b plasmid (B) show that rescuing caveolar disruption negates the depletion of caveolae which keeps CRFB1 molecules clustered under normal conditions. When Cav-1b is depleted and caveolae are not maintained with crosslinking reagent or cav-1b plasmid, minimal MxA expression is measured. The knockdown of CRFB1, CRFB2, and CRFB5 is the negative control; data indicate that there is low induction of MxA without IFN receptor subunits. When Cav-1b is depleted and CRFB1 is kept clustered, MxA expression remains at the same level as in the controls, demonstrating that the clustering of the receptor is essential for downstream signaling and that caveolin-1 plays a critical role in keeping the receptor clustered. Representative of 3 experiments; error bars indicate SEM (*, p<0.05). These results indicate dampening of MxA transcript production between control MO and Cav-1b MO prior to rescue of caveolae domains, and no significant difference of MxA transcript levels between Control MO and Cav-1b MO after rescue of caveolae domains.

A parallel experiment was conducted in ZFL cells that were transfected with control MO, Cav-1b MO, or CRFB1/CRFB2/CRFB5 MO (all subunits of the IFN-R) in order to measure the induction of antiviral genes downstream from the IFN-R. Polyinosinic-polycytidylic acid (poly(I:C)) was used in another experiment to mimic an infection and to stimulate the production of IFN by the immune system. Poly(I:C) is a synthetic analog of double stranded RNA (dsRNA) which is associated with viral infection. It is recognized by pattern recognition receptors (PRR) [45], [46] and leads to the induction of type I IFN and inflammatory cytokines. Cells were crosslinked with BS^3^, exposed to poly(I:C), or both crosslinked with BS^3^ and exposed to poly(I:C) (Figure 6a). Depletion of Cav-1b using the MO in ZFL cells is demonstrated in Figure S5. Time points shown correlate with the time of crosslinking (6 h post transfection) and the antiviral myxovirus resistance gene (MxA) measurement (30 h post transfection).

An additional experiment was performed with cells transfected together with both MO and cav-1b plasmid to rescue the effect of the Cav-1b knockdown. Cells were subsequently infected with SHRV (Figure 6b). Transcripts of MxA were measured by quantitative RT-PCR. MxA was chosen because its transcripts are produced solely from the IFN-α/β pathway and not the IFN-γ pathway [25]. As expected, upon either poly(I:C) exposure (Figure 6a) or SHRV infection (Figure 6b) Cav-1b MO samples displayed decreased MxA expression compared to controls (second group of bars, gray), as did CRFB1/CRFB2/CRFB5 MO samples (second group of bars, black). To rescue the effects of Cav-1b depletion, cells were also transfected with cav-1b plasmid which resulted in detection of low levels of MxA in the absence of SHRV (Figure 6b). Cells were then infected with SHRV and MxA gene expression was measured. With either crosslinking or cav-1b rescue, MxA gene expression remained comparable to that of control cells (Figure 6a and b, fourth group of bars in each, gray bars). We have thus demonstrated that when the depletion of Cav1b is rescued, the expression of MxA did not decrease.

## Discussion

By developing a more thorough understanding of the mechanisms of the antiviral immune response, we hope to find clues that will aid the development of new therapeutics and vaccine adjuvants capable of augmenting the immune system and providing more effective protection to the host. This study demonstrates an entry-independent mechanism for virus evasion of host cell defenses through disruption of clusters of signaling molecules organized within cav-1b-containing membrane domains. Upon viral infection, Cav-1b was downregulated (Figure 2a and d), leading to a decrease in the number of cav-1b-containing membrane domains [2]. We report here the first nanoscale visualization of CRFB1 association with cav-1b-containing membrane domains in intact cells and demonstrate the dramatic effect that depletion of cav-1b-containing membrane domains has on the antiviral response. The use of FPALM enabled imaging of the clustering and subsequent dispersal of CRFB1 following Cav-1 knockdown.

The primary focus of the present studies was to investigate the potential abrogation of the antiviral response. The data show that in Cav-1b knockdown cells, CRFB1 molecules are dispersed and cav-1b-containing membrane domains are disrupted during viral infection, leading to impairment of the antiviral response. This suggests that intact caveolin domains may be crucial for proper clustering and function of CRFB1. Receptor dispersal from Cav-1 knockdown suppressed the antiviral immune response by disrupting downstream signaling, indicating that CRFB1 organization within cav-1b-containing membrane domains is critical for IFN-mediated antiviral defense. The functional consequences of cav-1 depletion were shown by direct observation with FPALM of CRFB1 clustering and identification of the membrane protein responsible for maintaining this clustered state.

The CRFB1 subunit of the zebrafish IFN receptor complex has been reported to heterodimerize with CRFB5 [25]. Levraud *et al*. [25], assessed several candidates of the CRFB family as likely members of the IFN receptor complex and found that knockdown of CRFB1 and CRFB5 have a dramatic effect on zebrafish IFN responsiveness. The authors postulated that the two should be designated as the heterodimer subunits of the IFN receptor. We have considered this interesting question in light of our current findings. We would like to know where CRFB5 is localized and whether or not this receptor subunit also clusters or relies upon cav1b-containing domains for a robust IFN response. Such tantalizing questions are currently being investigated.

Type I IFN belongs to a class of cytokines that play a crucial role in the innate immune response to viral infection [47], [48]. Molecular patterns such as viral double stranded RNA are detected by PRR [49], [50], resulting in production of IFN and antiviral proteins. In zebrafish, as in mammals, IFN molecules interact with the IFN-R subunits [50], [51], which exist as heterodimeric complexes [24], [25]. The janus kinase and signal transducer and activator of transcription (JAK-STAT) signaling pathway is highly conserved evolutionarily, and it is believed that in zebrafish, Stat transduces signals through a classical JAK-STAT pathway [52]. Briefly, the JAK-STAT pathway becomes active upon IFN binding to IFN-R, but two discrete IFN pathways activate JAK-STAT: IFN-α/β and IFN-γ. It is possible to measure components upstream from ISRE or MxA, such as STAT phosphorylation, but nonspecific contributions from the IFN-γ pathway can occur, making it difficult to discriminate between the JAK-STAT contributions of the two pathways. It has been hypothesized that the IFN-γ response is attenuated due to reduced levels of Stat1 in the IFN-R knockout [53]. To investigate whether a reduced level of *Stat1* gene expression also contributed to a dampened IFN response in our current studies, *Stat1* transcripts were measured by qRT-PCR after Cav-1b depletion. A decrease in *Stat1* gene expression was observed at 24 hpi (Figure 3a) as a result of Cav-1b depletion. We further demonstrate that CRFB1 is dispersed and that downstream MxA signaling can be restored by maintaining CRFB1 clusters (Figure 6). This indicates that the clustering of CRFB1 is critical for an antiviral response. Cav-1b-containing membrane domains appear to corral the receptor molecules, thus providing an environment conducive to efficient signal transduction.

Previous studies in murine embryonic fibroblasts demonstrated that type I IFN receptors (IFN-R) and type II IFN receptors (IFNGR1 and IFNGR2) were associated with caveolae domains after DRM isolation [21]. In contrast, IFNAR and IFNGR distribution in HeLa cells showed that only IFNGR complexes could be found in DRMs after stimulation [54]. The significance of protein associations with lipid rafts must therefore be re-evaluated and interpreted with caution. In our studies, we use ZFL cells, in which the endogenous expression levels of cav-1b have been confirmed (data not shown). Localization of IFN receptors in cav-1b-containing membrane domains by microscopy is likely to yield less equivocal results than biochemical DRM isolation. Our study yields images through the use of FPALM, which circumvents the resolution limit imposed by optical diffraction in conventional light microscopy.

Membrane structure and organization are important for many signaling processes. In this study, CRFB1 clustering in cells was examined and the results provided insights into the dynamic behavior of this receptor. Super-resolution imaging with FPALM showed that CRFB1 clustering is mediated by cav-1b-containing membrane domains and that disruption of such domains results in dispersal of receptor molecules. The consequence of CRFB1 dispersal was a dampened antiviral response. Studies were performed using either poly(I:C) stimulation or SHRV infection, both of which will induce IFN, the ligand that will bind to CRFB1 and stimulate it. It is important to note that the crosslinking reagent is non-specific and may impact cellular function because it crosslinks everything on the cell surface, not just cav-1 or CRFB1 molecules. We tested for non-specific crosslinking effects by also depleting CRFB1/2/5 and demonstrated that nonspecific induction of MxA due to crosslinking of other cellular components does not occur. We also rescued CRFB1 clustering through exogenous plasmid expression of cav-1b and found that after SHRV infection, expression levels of MxA remained essentially equal to controls (Figure 6b), similar to results seen in Figure 6a. MxA was measured because it is a selective and quantitative indicator of antiviral activity that is produced through induction of the IFN pathway. Taken together our data demonstrate that viral infection is exacerbated due to the reduced ability of Cav-1b morphant embryos to clear the infection resulting from the dispersal of CRFB1 and subsequent decrease in *Stat1* gene expression, ISRE activation, and MxA induction.

Our results contrast with the conclusions of many studies demonstrating that viruses use caveolae as a method of entry [55], [56], [57], but not as a means to alter the host immune response. Others have observed virus infections that do not use caveolae as a method of entry [13], [14], [15], [17], but did not necessarily study the role of caveolae in the antiviral immune response. We took a novel approach and discovered that SHRV downregulates Cav-1 expression to disrupt the host antiviral response. Many viruses in a range of species have developed mechanisms to target and evade the IFN system [58], [59], [60]. The studies outlined here reveal that viruses can escape the antiviral immune response by downregulating cav-1b protein levels, leading to a disruption of antiviral signaling through dispersion of IFN-R and abrogation of downstream signal transduction. We assessed the virus induced downregulation of cav-1b compared to the morpholino depletion of cav-1b and found that viral infection alone is enough to decrease cav-1b protein levels and dampen ISRE activity (Figures 2d and 3, respectively). Taken together, these studies support the hypothesis that cav-1b-containing membrane domains provide the local environment for interaction of critical antiviral receptor molecules. Additionally, these studies have demonstrated that Cav-1b is responsible for maintaining CRFB1 clusters and have shown the functional consequences of Cav-1 knockdown. From these observations, it is postulated that cav-1b-containing membrane domains increase CRFB1 signaling efficiency by concentrating receptor molecules so that proteins remain at the site of signaling. There have been several studies of immunity in zebrafish that demonstrate similarities to immune function in higher vertebrates [22], [23], [27], [28], [42], [61]. In addition, a publically available microarray database (European Bioinformatics Institute’s Gene Expression Atlas, part of the European Molecular Biology Laboratory; http://www.ebi.ac.uk/gxa/) was used to identify downregulation of cav-1 in humans after infection with viruses such as HSV and HIV. Our identification of a cav-1 binding domain in human IFN-R, together with the high degree of functional conservation between the immune system of zebrafish and higher vertebrates, suggests that our studies are relevant to immunity in higher vertebrates. FPALM studies provided critical insight into the mechanisms of viral evasion and modulation of membrane domains that are critical to the host immune response to virus infection. Understanding the complex mechanisms through which viruses modulate immune function should provide insight into a range of potential targeted antiviral therapies.

## Materials and Methods

### Ethics Statement

Zebrafish used in this study were handled in accordance with the recommendations in the Guide for the Care and Use of Laboratory Animals of the National Institutes of Health. The protocol was approved by the Institutional Animal Care and Use Committee (IACUC) at the University of Maine (Protocol Number: A2008-06-03). IACUC approved guidelines for zebrafish care were followed using standard procedures (www.zfin.org).

### Cell Culture, Constructs, and Transfection

#### Cell culture

EPC (epithelioma papulosum cyprinid) cells originated from carp epidermal herpes virus-induced hyperplastic lesions [62]. EPC cells have a broad sensitivity for fish viruses and are commonly used for isolation, propagation, and diagnostic assays for fish viruses. EPC cells were maintained at 28°C, 4% CO_2_ in Minimum Essential Medium (MEM) (GIBCO-Invitrogen, Carlsbad, CA) supplemented with 10% heat-inactivated fetal bovine serum (GIBCO-Invitrogen, Carlsbad, CA) and antibiotics.

ZFL (zebrafish liver) cells were derived from normal adult zebrafish liver [63]. They display an epithelial morphology. Ghosh *et al* demonstrated that the cells exhibit properties in culture that are associated with liver cell function *in vivo*. ZFL cells were maintained at 28°C, 0% CO_2_ in LDF culture medium (50% Leibovitz’s L-15 Medium, 35% Dulbecco’s modified Eagle’s Medium, and 15% F-12 Medium) supplemented with heat-inactivated fetal bovine serum.

#### Expression plasmids

A modified pEGFP-N1 plasmid (Clontech) containing PA-mCherry in place of mEGFP [64] was digested with XmaI and NotI (New England Biolabs) to linearize the plasmid. *Cav-1b* was cloned from a 30dpf zebrafish cDNA library and PstI and XmaI sites were added by polymerase chain reaction. Dendra-CRFB1 was generated using a dendra2-HA construct [65] in which PstI and XmaI restriction sites were added to *CRFB1* by polymerase chain reaction. *CRFB1* was subsequently inserted between PstI and XmaI, replacing HA from the vector. The final constructs were purified by Endotoxin Free Miniprep (Omega).

#### Cell transfection

ZFL cells were transfected by nucleofection according to the manufacturer’s protocol (Lonza). Cells were transfected 24 h prior to fixation. For fixation, cells were removed from the incubator and rinsed three times in Dulbecco’s PBS (BioWhittaker Lonza, Walkersville, MD), and incubated at room temperature for 20 min in 4% paraformaldehyde (Sigma-Aldrich). Immersion water and PBS were both irradiated for ∼15 min by 500 W UV-lamp to reduce background fluorescence. During measurements, UV-bleached Dulbecco’s PBS was used as the imaging medium.

#### Luciferase assay

Luciferase assays were performed in a manner similar to that described previously [22], [44]. The IFN stimulated regulatory element (ISRE)-reporter vector ISRE-luc was provided by R. Medzhitov (Yale University, New Haven, CT) [44]. Prior to transfection, cells were allowed to reach 70–80% confluence in a T75 flask, at which point they were resuspended in buffer SF (Lonza) at 4×10^5^ cells/20 µL and mixed with a total of 250 ng of indicated plasmid DNA, 250 of either the pB2×-luciferase or pGL3-IFN reporter construct, and 6.25 ng of *pRL-CMV Renilla* luciferase internal control construct. Cells were then electroporated using the Amaxa 96-well shuttle (Lonza) using program EW-158. Cells were then plated at 1×10^5^ cells/well, in triplicate, using fresh medium and incubated at 28°C for 30 hours prior to 0.5 µg/ml polyinosinic-polycytidylic acid (poly(I:C)) exposure for 6 hr. Since poly(I:C) resembles the RNA of infectious viruses, it was used to mimic an infection and stimulate the immune system to produce IFN and other cytokines. Following poly(I:C) exposure or SHRV infection, cells were lysed and firefly and *Renilla* luciferase activity was measured using the Dual-Luciferase reporter assay system (Promega). Two experiments were performed with three replicates per experiment. The mean of the three replicates was taken for each experiment, and the standard deviation of the means was taken to generate the SEM. Relative luminescence units (RLU) were measured in a GLOMAX Luminometer (Promega).

### Zebrafish and Morpholino Microinjections

#### Zebrafish care and maintenance

Wild-type (strain AB) fish were maintained in the Zebrafish Facility at the University of Maine, Orono. The zebrafish facility is maintained according to the Institutional Animal Care and Use Committee (IACUC) standards. Fertilized eggs were collected in petri dishes at the one-cell stage before the start of experiments and raised in egg water (60 µg/ml Instant Ocean sea salts) at 28°C.

#### Microinjection of oligonucleotide morpholinos

Antisense morpholino oligomers (MOs) were designed and synthesized by Gene Tools, LLC (Eugene, OR). The MOs were diluted for injection in 1× Danieau solution (58 mM NaCl, 0.7 mM KCl, 0.4 mM MgSO4, 0.6 mM CA(NO3)2), 5 mM HEPES (ph7.6), with phenol red as indicator. For all MO experiments, the standard control MO was used from Gene Tools, LLC, and has the following sequence: 5′-CCTCTTACCTCAGTTACAATTTATA-3′. For all injections, the injection volume was 3 nl.

The translation blocking cav-1 MOs were previously published by Fang [2] and are targeted to the ATG start sites of cav-1a and cav-1b mRNAs. Cav1 MOs and control MO were injected at the same concentration. The Cav-1a MO sequence is 5′-TCCCGTCCTTGTATCCGCTAGTCAT-3′ and the cav-1b MO sequence is 5′-TTCGTTGATGCTGTCGTTATCCATT-3′. MOs were microinjected into zebrafish embryos at 6 ng/embryo during the 1–2 cell stage. Injected embryos subsequently developed in egg water at 28°C.

All CRFB MOs were previously published [24], [25]. CRFB1 MO is a translation blocking MO with the sequence 5′-CAGTGTATGATGATGATGTCTTCAT-3′. CRFB2 MO is a splice blocking MO with the sequence 5′-CTATGAATCCTCACCTAGGGTAAAC-3′. CRFB5 MO is a translation blocking MO with the sequence 5′-CAGGGCACACTCCTCCATGATCCGC-3′.

### Virus and Viral Burden Assays

Snakehead rhabdovirus (SHRV) was propagated in EPC cells. Cells were infected at a multiplicity of infection (MOI) of 0.1. The supernatant was then collected and filtered to obtain purified virus at a titer of 3.16×10^7^ 50% tissue culture infectious doses (TCID_50_)/ml. For infection and imaging experiments, cell monolayers at ∼70–80% confluency were infected 24 h prior to fixation and imaging. SHRV infection at 0.1 MOI proceeded for 1 h at 28°C before cells were overlain with additional growth medium for another 23 h (24 h total infection time).

Wild-type and caveolin-deficient zebrafish embryos were infected by static immersion 48 hours post fertilization (hpf) for 5 hours with 1×10^6^ TCID_50_/ml SHRV or maintained as uninfected controls. Twenty fish were collected at 24 hr post infection (hpi) for each treatment and homogenized in minimum essential medium (GIBCO-Invitrogen, Carlsbad, CA), with 50 µg/ml gentamycin. The homogenate was frozen at −80°C before the TCID_50_ assay.

TCID_50_ is a type of virus quantification method. This endpoint dilution assay enables us to determine how much virus is needed to produce a pathological change (observed as cytopathic effects, or CPE) in 50% of inoculated cells in culture. CPE (i.e. infected cells) was manually observed and recorded for each virus dilution. For our experiments, supernatants previously frozen at −80°C were thawed to be used in TCID_50_ assays and subsequently monitored for cytopathic effects (CPE). After seven days, CPE was determined and the TCID_50_/ml of the virus was calculated according to the Reed-Muench formula [66].

Virus infection in cell culture experiments was also performed with SHRV propagated in EPC cells. For these studies, cells were infected at an MOI of 0.01. The virus was allowed to adsorb for 1 h. Subsequently, virus was removed and regular cell culture media was replaced. Dual luciferase assays were performed as described after 24 hpi.

### RNA Extraction, cDNA Synthesis, and Quantitative Real-time PCR

Total RNA was extracted after Cav-1b MO-injected and control MO injected fish were infected with SHRV by static immersion for 5 hr. Viral samples were collected at 24, 48, and 72 hpi by homogenizing 10 fish from each treatment, per time point in TRIzol reagent (Invitrogen, Carlsbad, CA) and subsequently stored at −80°C. RNA was extracted according to the manufacturer’s protocol. Reverse transcription reactions were performed as previously described [38] to synthesize cDNA. Quantitation of MxA was carried out using an I-cycler IQ Detection System (Bio-Rad Laboratories, Hercules, CA). The cycling parameters used were chosen as described previously [38]. Fluorescence measurements were made at each cycle during the annealing step and the copy number was determined based on a standard curve using the iCycler software. The value for each sample was normalized to the corresponding β-actin value to determine relative copy number. Fold inductions were calculated by dividing the copy number in the virus infected samples by the uninfected samples at the same time point.

To identify the cell lineages in which cav-1 is expressed, zebrafish tissues were dissected into Trizol (Invitrogen, Carlsbad, CA) and total RNA was purified in preparation for qPCR. Lymphoid and myeloid cells (frozen pellets) isolated from zebrafish kidneys and purified by fluorescence-activated cell sorting were generously provided by Dr. David Traver (University of California, San Diego, CA) [28], [67], [68] and resuspended in Trizol for RNA purification. Total RNA from tissues (2 ug) and sorted cells (1 ug) was reverse transcribed (SuperScript™ III Reverse Transcriptase, Invitrogen) and subjected to thermal cycling with gene-specific primers and TITANIUM™ Taq DNA polymerase (Clontech, Mountain View, CA). Expression of cav1a and cav1b isoforms was detected using primers previously described [2] and 40 cycles with an annealing temperature of 70°C. PCR conditions and primer sequences for detecting myeloperoxidase (mpx), TCRa and β-actin expression were described previously [69]. Liver tissue was isolated for detection of *cav-1b, CRFB1, L-FABP,* and *β-actin* expression in the liver of a 48 hpf embryo according to previously published methods [70]. Total RNA from liver tissue was extracted and cDNA synthesized as described above. PCRs were analyzed by gel (2% agarose) electrophoresis.

### Lysate Preparation and Immunoblotting of Zebrafish Embryos and ZFL Cells

Embryos were prepared in a manner similar to that published previously [2]. Embryos were collected, egg water removed, and flash-frozen in a slurry of dry ice prior to storage at −80°C. For use, frozen embryos were solubilized in RIPA lysis buffer (Pierce, Rockford, IL) and HALT protease/phosphatase inhibitor cocktail (Thermo Scientific). Embryonic zebrafish were incubated on ice for 30 minutes prior to centrifugation at 18,000×g for 15 minutes at 4°C. Supernatants were collected as whole cell lysates.

ZFL cells were transfected with control morpholino (MO) or Cav-1b MO to knock down the expression of Cav-1b. Samples were taken at 6 and 30 h post transfection (hpt), corresponding to the time points used to perform experiments shown in Figure 6. For sampling, cells were centrifuged at 90×g for 10 minutes at 4°C. Cells were washed twice in DPBS (BioWhittaker Lonza, Walkersville, MD) prior to storage at −80°C. For use, cell pellets were solubilized in RIPA lysis buffer (Pierce, Rockford, IL) and HALT protease/phosphatase inhibitor cocktail (Thermo Scientific). Cells were incubated on ice for 30 minutes prior to centrifugation at 3500×g for 10 minutes at 4°C. Supernatant was collected for use as the soluble fraction.

To determine protein concentrations, a Bradford assay was performed using the Bio-Rad Protein Assay Dye Reagent (Bio-Rad Laboratories, Hercules, CA). Equal volumes of total cell lysate were solubilized in lysis buffer, boiled for 5 minutes, and fractionated by SDS-PAGE Gel electrophoresis. Fractionated proteins were transferred to a nitrocellulose membrane by electrophoresis, blocked with 5% non-fat dry milk, and immunoblotted with the anti-human Cav-1 polyclonal antibody (1∶500 dilution, BD Transduction Laboratories). Cav-1 protein was visualized using horseradish peroxidase conjugated secondary antibody (Santa Cruz Biotechnology) and the Supersignal Chemiluminescence System (Pierce, Rockford, IL). Membranes were re-probed with antibody against β-actin to control for protein loading.

### Cross-linking Experiments with BS^3^ Reagent

Cells were transfected via nucleofection with control MO, Cav-1b MO, or combined CRFB1/CRFB2/CRFB5 MO and allowed to recover/adhere to cell culture plates for 6 hr prior to bis(sulfosuccinimidyl)suberate (BS^3^) cross-linking treatment. Cross-linking reactions were performed according to the manufacturer’s procedures (Pierce, Rockford, IL). Cells were subsequently washed 3 times with 500 µl DPBS and replenished with media and returned to the incubator for 24 hr post exposure (hpe) to BS^3^. For cells that were exposed to 1 µg/ml poly(I:C) (Invitrogen), treatments were initiated 18 hpe to BS^3^ reagent and proceeded for 6 hr. Following exposures, RNA samples at sequential time points were taken with TRIzol according to the manufacturer’s procedures. RNA extractions, cDNA synthesis, and quantitative RT-PCR were performed as described above.

### Single-molecule Microscopy (FPALM)

In normal fluorescence microscopy, many of the fluorescent molecules are visible at the same time and their images are blurred together by diffraction. Since diffraction blurs objects smaller than 200–250 nm, important biological details can be obscured. FPALM circumvents diffraction by limiting the number of visible/fluorescent molecules visualized at once. Rather, many small subsets of fluorescently labeled molecules within a sample are imaged separately, such that each molecule is distinct. This is achieved by optical control of molecular transitions between bright and dark states. By limiting the numbers of emitting molecules and activating a subset of molecules, and then imaging and photobleaching them and repeating this process for many subsets of molecules, coordinates of thousands of molecules can be obtained [31], [32]. This iterative process is repeated until sufficient molecules have been localized and the structure of the sample is revealed. The positions of the single molecules can be determined (localized) with a precision better than diffraction limited resolution. The FPALM image is generated by plotting the positions of the localized molecules.

#### Single color FPALM imaging and analysis

Single color FPALM imaging and analysis were performed as described earlier [31], [32], [33]. A 405 nm diode laser (BCL-405-15, Crystalaser,Reno, NV) was used to activate labeled molecules in the sample, while a 556 nm (LRS-556-NM-100-10, Laserglow, Toronto, Canada) diode laser was used to read out active molecules. Both beams were focused at the back aperture of a 60×/1.2NA water-immersion objective lens (UPLAPO60×W, Olympus, Melville, NY) to produce widefield illumination at the sample. Fluorescence from the sample was collected by the objective, separated from laser light by a dichroic mirror (T565LP, Chroma Technology, Rockingham, VT), bandpass filtered (ET605/70M, Chroma), and imaged by an EMCCD camera (iXon+ DU897DCS-BV, Andor Scientific, South Windsor, CT) operated at an EM gain of 200 and frame rate of ∼31.5 Hz.

The camera was controlled using Solis software (Andor). Additional achromatic lenses (f = +60 mm and f = +200 mm, Newport Corporation, Irvine, CA), arranged as a telescope, were mounted in the detection path to provide additional magnification and to produce an effective camera pixel size of ∼136 nm. A motorized filter wheel (FW102, Thorlabs, Newton, NJ) containing neutral density filters provided control over the activation intensity to maintain a density of visible molecules of ∼1 per µm^2^ or less.

Cells were selected for FPALM imaging by exciting (475/40×, Chroma) the sample with an Hg lamp and searching for green fluorescence (bandpass-filtered, HQ535/50M, Chroma) to locate cells transfected with CRFB1-dendra2. During post acquisition analysis, each frame of an image series (typically 10,000 frames) was background subtracted and positive intensity peaks with at least one pixel above a minimum threshold were fitted to a two-dimensional Gaussian to determine the x and y coordinates, amplitude (I_0_), e^2^ radius (r_0_), and an offset. Fitted values of I_0_ and r_0_ were then used to calculate the number of detected photons. Fits that yielded N and r_0_ consistent with that expected for a single molecule were recorded for further analysis. For each localized molecule the localization precision was calculated using the standard analytical equation from the literature, including an additional 30% [71]. Lateral drift of the sample stage has been characterized previously [33] and was assumed to be negligible over the duration of these experiments, compared to the estimated lateral resolution of ∼10–30 nm. All analysis was performed using custom software written in Matlab (Mathworks, Natick, MA).

#### Two-color FPALM imaging and analysis

Two-color imaging of fixed ZFL cells transfected with PAmCherry-cav1 and dendra-CRFB1 was performed at room temperature using the geometry employed in [72]. A dichroic mirror (Z568RDC, Chroma) and emission filters (FF01-630-92-25, Semrock and ET605/70M, Chroma for transmitted and reflected wavelengths, respectively) were mounted in the detection path between the dichroic mirror and the electron multiplying charge-coupled device camera (EMCCD) (iXON+DU897DCS-BV, Andor Technology, South Windsor, CT). Illumination of the sample was achieved by placing a lens f = +350 mm) (Thorlabs, Newton, NJ) near the rear epi-illumination port of an inverted microscope (IX71, Olympus America, Melville, NY) to focus the beams to the secondary (back) focal plane of a 60×, 1.2 NA water-immersion objective lens (UPLAPO60×W, Olympus). A 405 nm diode laser (BCL-405-15, Crystalaser) was used for photoactivation and a 556 nm laser was used for readout. Frames were acquired at 31.5 Hz (EM gain 200) with the EMCCD camera. Images were acquired using Labview software (National Instruments Corporation, Austin, TX).

Analysis for two color imaging was an extension of standard FPALM analysis, described above and previously reported [32], [73]. Analysis was performed using MATLAB software (Mathworks, Inc. Natick, MA) as follows: raw frames containing the two spatially separated images were background subtracted, then correlated and superimposed for localization. Each localized molecule is identified by α, the ratio of emission in the red detection channel divided by the sum of the intensity in both red and yellow channels [74]. The species of each localized molecule was assigned as either dendra2-CRFB1 (or dendra2-SHRV for Figure S4A) or PAmCherry-caveolin using a range of α values determined numerically (typically 0.55–0.64 for dendra2 and 0.68–0.75 for PAmCherry), such that the error in assignment to either species was <5%.

#### Pair correlation calculations

Pair correlation analysis and calculation was performed similar to methods previously described by the Hess laboratory [73].

#### Single color pair correlation

Coordinates obtained from FPALM imaging were used to calculate pair correlation functions. Localizations of the same molecules in consecutive frames were removed from the data set by linking molecules in the i^th^ frame to molecules (i+1)^th^ frame that were separated by less than 3 times the median localization precision. The positions of linked molecules were then averaged for use in pair correlation calculations. Values of g(r)>1 indicate correlation between species while g(r)<1 indicates anti-correlation. For uniform distribution of molecules, g(r) = 1 is expected. Calculated values of g(r) were fitted to the analytical correlation function [75], including a constant offset, where A is the amplitude and r_0_ is the correlation length, and η is a number.

#### Two color pair correlation

Prior to pair correlation calculation using coordinates obtained from two-color FPALM analysis, duplicate localizations of the same molecule in consecutive frames were removed as described above. The cross-correlation, g(r), of species A with species B was calculated from:
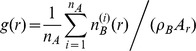
Where 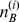
(r) is the number of molecules of species B that lie within a Δr = 10 nm-thick circular shell of radius r from the i-th molecule of species A, A_r_ is the area of the shell of radius r±(Δr/2), n_A_ is total number of species A used in the summation over index i, and ρ_B_ is the average density of species B. The summation was performed only over molecules of species A that were more than a distance d from the edge of the cell and the imaged region of interest, where d is the maximum length scale of interest for pair correlation analysis such that edge corrections were not required.

## Supporting Information

Figure S1 **Principles of FPALM.** By limiting the number of fluorescent molecules visible at once, the images of the individual molecules become distinguishable. (A) Molecules are initially in an inactive (non-fluorescent) state. (B) Sparse subsets of molecules are converted into a fluorescent state by the activation beam (purple) when excited by the readout laser (green) and are imaged (C) until deactivated or photobleached (D). Molecules are localized by fitting the image with a two-dimensional Gaussian. Cycles of activation (B,E), readout and localization (C,F), and photobleaching (D,G) are repeated for many subsets of molecules. Rendered images with few (H) and large number (I) of localized molecules show buildup of structural detail as density increases. (J) Conventional image with diffraction-limited resolution. *Image from: Localization-Based Super-Resolution Light Microscopy, by Kristin A. Gabor, Mudalige S. Gunewardene, David Santucci and Samuel T. Hess. Microscopy Today, Volume 19, Issue 04 (Jul 2011), pp. 12–16. Copyright ©2011 Microscopy Society of America. Reprinted with the permission of Cambridge University Press.*
(TIF)

Figure S2 **Caveolin-1 Expression in Cell Culture and Tissue-Specific Zebrafish cDNA Pools.** A) qPCR demonstrates expression of endogenous *cav1b* and *CRFB1* transcripts in RNA isolated from cultured ZFL cells. B) PCR was performed in liver tissue isolated from zebrafish embryos at the stage of virus infection (48 hpf) and demonstrates the expression of cav1b (338 bp), CRFB1 (201 bp), L-FABP (265 bp) and B-actin (301 bp) in the liver tissue of zebrafish embryos. C) PCR was performed to detect *cav-1a and cav-1b* gene expression in cDNA pools isolated from specific zebrafish tissues. Of note, *cav-1b* expression was detected in the kidney, lymphocyte, and myeloid lineages.
(TIF)

Figure S3 **Cav-1a is also modulated as a result of SHRV infection.** A) Quantitative RT-PCR results revealed fold changes in the expression levels of *Cav-1a* in infected embryos when compared to uninfected embryos. Zebrafish were exposed seven days post fertilization (dpf) to 1×10^6^ TCID_50_/mL virus. Total RNA was isolated from at 12, 24, and 48 hours post infection and reverse transcribed to cDNA (n = 20 fish per time point). Error bars represent SEM for three replicates. B) Zebrafish embryos were injected with Control MO or Cav-1a morpholino (MO) to knock down the expression of Cav-1a. Fish were infected 48 hpf with 1×10^6^ TCID_50_/ml virus and monitored for mortality. Results are representative of three separate experiments. Statistical analysis (Wilcoxon test) of the Kaplan-Meier curve was performed (*, p<0.05).
(TIF)

Figure S4 **SHRV does not utilize caveolae to enter the host cell.** FPALM imaging demonstrates no colocalization between fluorescently labeled virus and Cav-1b molecules early in the infection. Shown is a representative cell (total ≥8) of Cav-1b at 10 min post infection (A) and 2 h post infection (B). This indicates that SHRV does not use caveolae as a means of entry, suggesting that entry through caveolae will not be affected as a result of Cav-1 knockdown. For all images, 60×/1.2 NA. Scale bars, 1 µm.
(TIF)

Figure S5 **Knockdown of Cav-1b in ZFL cells.** Western blot showing efficacy of MO (morpholino) in ZFL cells. Cells were transfected with Control morpholino (MO) or Cav-1b MO to knock down the expression of Cav-1b. Samples were taken at 6 and 30 h post transfection (hpt), corresponding to the time points used to perform experiments shown in Figure 7. At 6 hpt there is a marked decrease in cav-1 protein expression compared to control cells, while at 30 hpt a slight decrease in cav-1 protein expression is still observed. Membranes were re-probed with antibody against β-actin to control for protein loading.
(TIF)
