# Supplementary figures and images for: Super Resolution Microscopy Reveals that Caveolin-1 Is Required for Spatial Organization of CRFB1 and Subsequent Antiviral Signaling in Zebrafish
Source: PLoS One. 2013 Jul 9;8(7):e68759. doi: 10.1371/journal.pone.0068759 (PMC3706321; doi:10.1371/journal.pone.0068759)

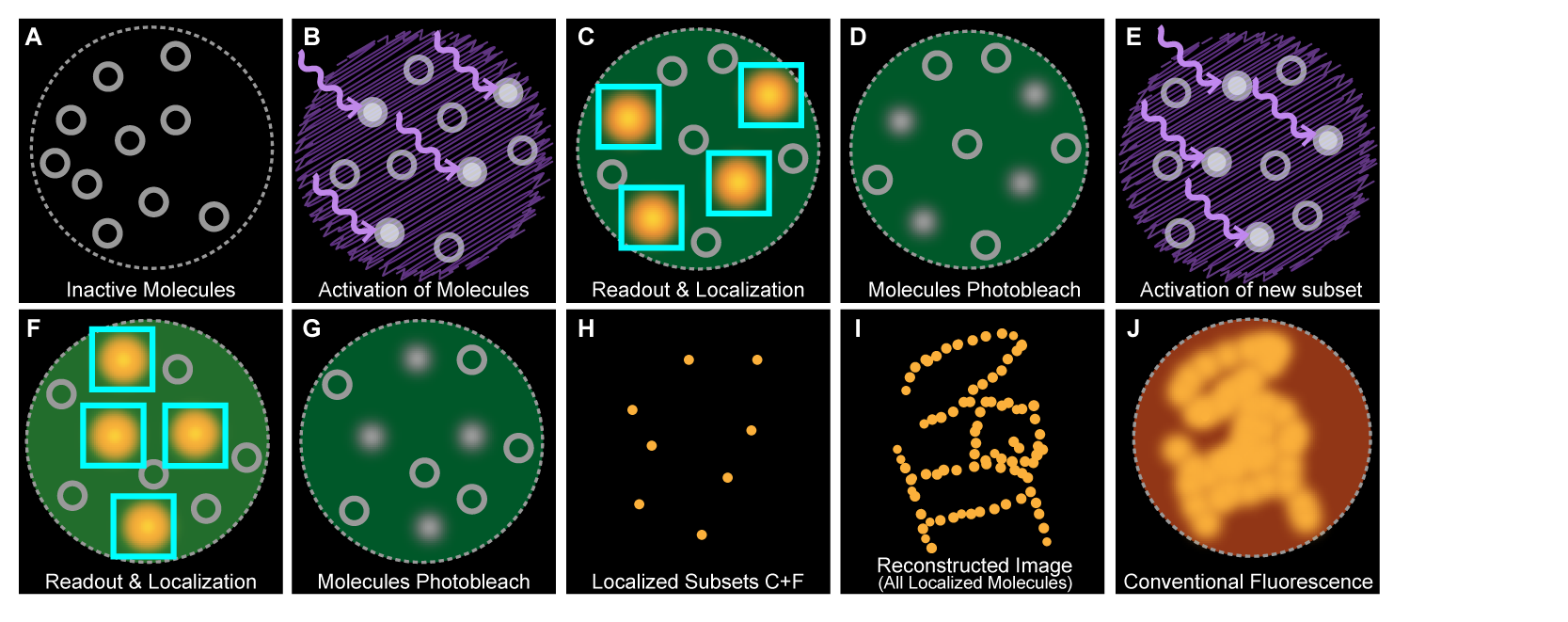

Supplement: Figure S1 — Principles of FPALM. By limiting the number of fluorescent molecules visible at once, the images of the individual molecules become distinguishable. (A) Molecules are initially in an inactive (non-fluorescent) state. (B) Sparse subsets of molecules are converted into a fluorescent state by the activation beam (purple) when excited by the readout laser (green) and are imaged (C) until deactivated or photobleached (D). Molecules are localized by fitting the image with a two-dimensional Gaussian. Cycles of activation (B,E), readout and localization (C,F), and photobleaching (D,G) are repeated for many subsets of molecules. Rendered images with few (H) and large number (I) of localized molecules show buildup of structural detail as density increases. (J) Conventional image with diffraction-limited resolution. Image from: Localization-Based Super-Resolution Light Microscopy, by Kristin A. Gabor, Mudalige S. Gunewardene, David Santucci and Samuel T. Hess. Microscopy Today, Volume 19, Issue 04 (Jul 2011), pp. 12–16. Copyright ©2011 Microscopy Society of America. Reprinted with the permission of Cambridge University Press. (TIF) [file pone.0068759.s001.tif]

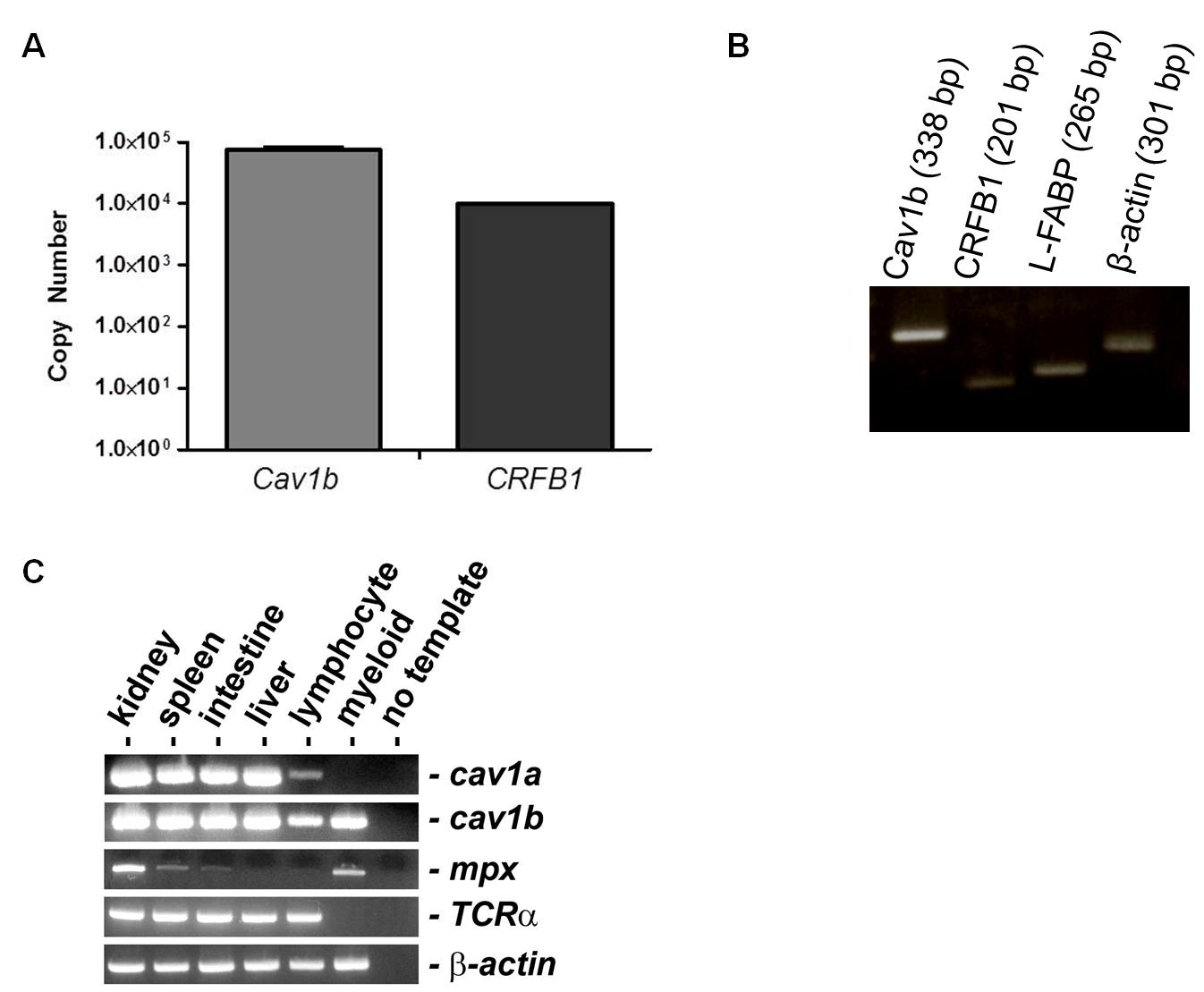

Supplement: Figure S2 — Caveolin-1 Expression in Cell Culture and Tissue-Specific Zebrafish cDNA Pools. A) qPCR demonstrates expression of endogenous cav1b and CRFB1 transcripts in RNA isolated from cultured ZFL cells. B) PCR was performed in liver tissue isolated from zebrafish embryos at the stage of virus infection (48 hpf) and demonstrates the expression of cav1b (338 bp), CRFB1 (201 bp), L-FABP (265 bp) and B-actin (301 bp) in the liver tissue of zebrafish embryos. C) PCR was performed to detect cav-1a and cav-1b gene expression in cDNA pools isolated from specific zebrafish tissues. Of note, cav-1b expression was detected in the kidney, lymphocyte, and myeloid lineages. (TIF) [file pone.0068759.s002.tif]

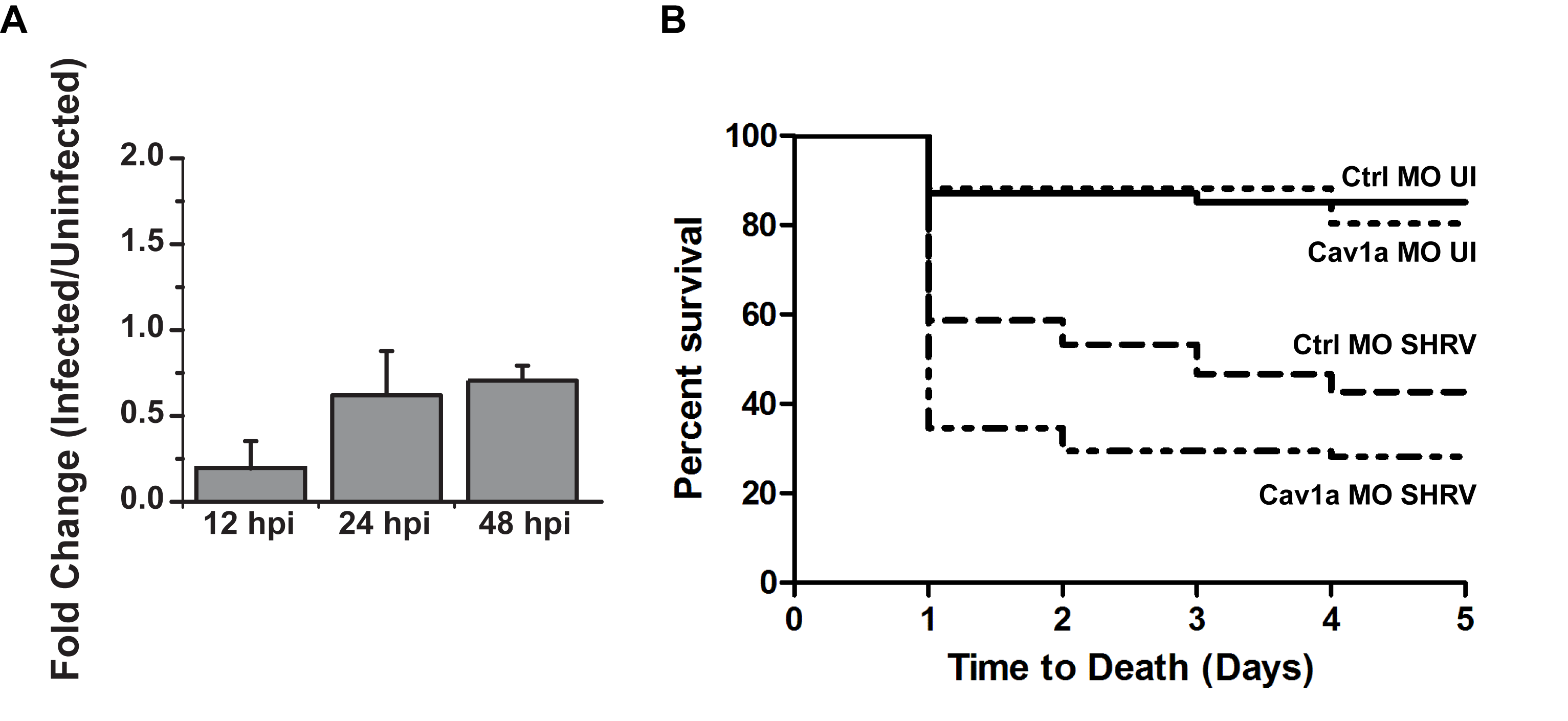

Supplement: Figure S3 — Cav-1a is also modulated as a result of SHRV infection. A) Quantitative RT-PCR results revealed fold changes in the expression levels of Cav-1a in infected embryos when compared to uninfected embryos. Zebrafish were exposed seven days post fertilization (dpf) to 1×10^6^ TCID50/mL virus. Total RNA was isolated from at 12, 24, and 48 hours post infection and reverse transcribed to cDNA (n = 20 fish per time point). Error bars represent SEM for three replicates. B) Zebrafish embryos were injected with Control MO or Cav-1a morpholino (MO) to knock down the expression of Cav-1a. Fish were infected 48 hpf with 1×10^6^ TCID50/ml virus and monitored for mortality. Results are representative of three separate experiments. Statistical analysis (Wilcoxon test) of the Kaplan-Meier curve was performed (*, p<0.05). (TIF) [file pone.0068759.s003.tif]

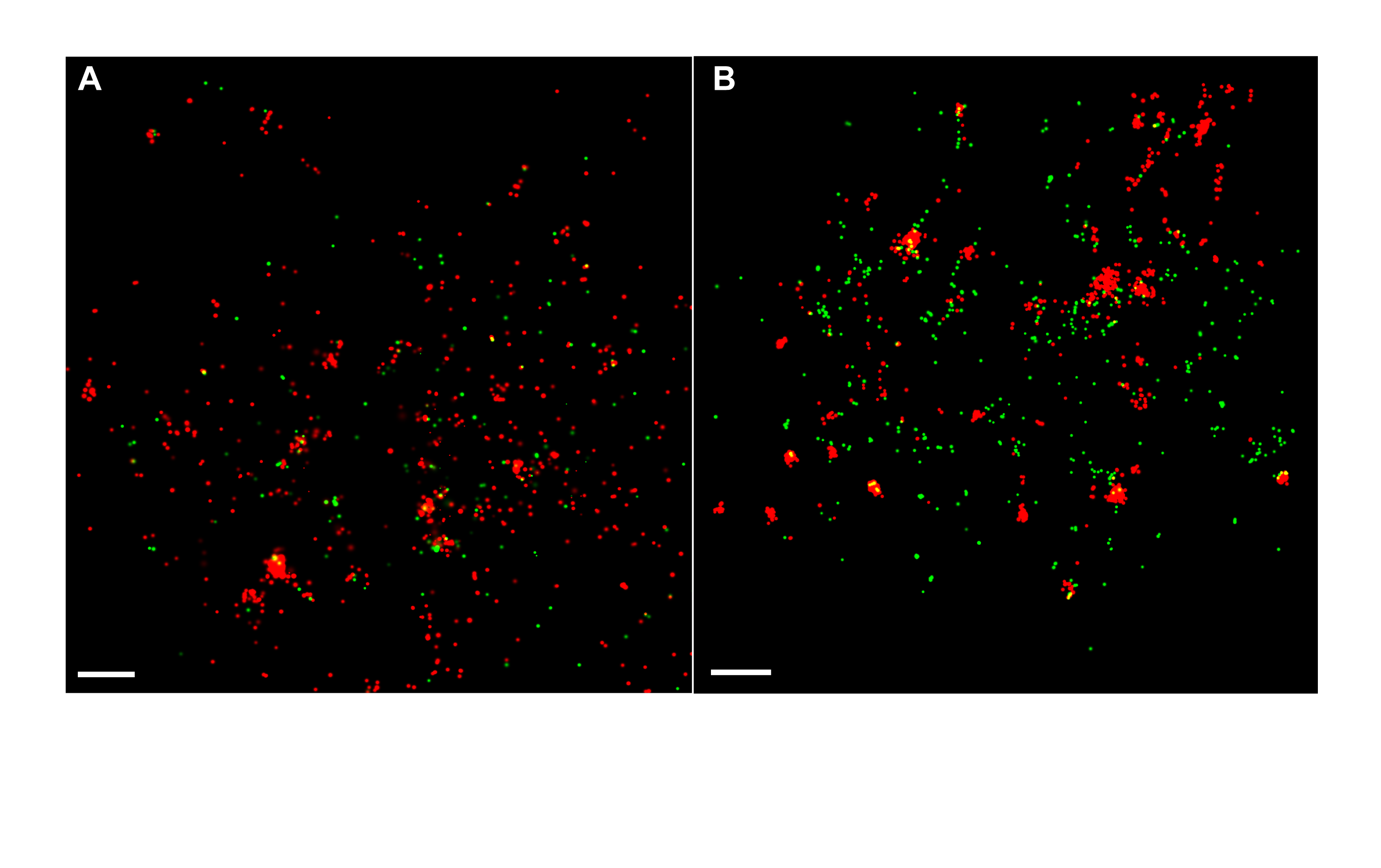

Supplement: Figure S4 — SHRV does not utilize caveolae to enter the host cell. FPALM imaging demonstrates no colocalization between fluorescently labeled virus and Cav-1b molecules early in the infection. Shown is a representative cell (total ≥8) of Cav-1b at 10 min post infection (A) and 2 h post infection (B). This indicates that SHRV does not use caveolae as a means of entry, suggesting that entry through caveolae will not be affected as a result of Cav-1 knockdown. For all images, 60×/1.2 NA. Scale bars, 1 µm. (TIF) [file pone.0068759.s004.tif]
